# A Novel Evaluation Method for Building Construction Project Based on Integrated Information Entropy with Reliability Theory

**DOI:** 10.1155/2013/573014

**Published:** 2013-02-28

**Authors:** Xiao-ping Bai, Xi-wei Zhang

**Affiliations:** School of Management, Xi'an University of Architecture and Technology, Xi'an Shanxi 710055, China

## Abstract

Selecting construction schemes of the building engineering project is a complex multiobjective optimization decision process, in which many indexes need to be selected to find the optimum scheme. Aiming at this problem, this paper selects cost, progress, quality, and safety as the four first-order evaluation indexes, uses the quantitative method for the cost index, uses integrated qualitative and quantitative methodologies for progress, quality, and safety indexes, and integrates engineering economics, reliability theories, and information entropy theory to present a new evaluation method for building construction project. Combined with a practical case, this paper also presents detailed computing processes and steps, including selecting all order indexes, establishing the index matrix, computing score values of all order indexes, computing the synthesis score, sorting all selected schemes, and making analysis and decision. Presented method can offer valuable references for risk computing of building construction projects.

## 1. Introduction

The evaluation decision of building construction schemes is a complex multiobjective and multifactor problem, selecting a reasonable goal structure system of evaluation schemes and the optimal scheme is very important in the building construction decision process [1].

Until now, there have been many references studying the optimization decision problem of building construction schemes, and many concepts about it have been set up. Among them, some research results only aim at the small range special field; for example, some researchers study pit bracing construction scheme decision problem [2]. Analytical Hierarchy Process (AHP) is a common method; however, its convictive power is not strong enough because it lacks quantitative data analysis [3–5]. In [6], authors integrated value engineering principle with technical and economic factors to make evaluating and decision making of construction schemes, its advantage is more considering evaluation factors included in construction schemes, but the selection of evaluation values is fuzzy [6]. In addition, grey correlation [7], minimum variance [8], fuzzy decision, projection pursuit, and other methods are also used in decision-making of project schemes [9, 10].

The optimization decision-making evaluation of construction schemes is a multiobjective process; many targets should be analyzed. Some references select cost, progress, quality, and reliability as evaluation targets, such as in [11, 12], analysis of time-cost-quality tradeoff optimization in construction project management is presented [11, 12], but on the whole there is a shortage of systematic deep discussion, and qualitative study is dominant. 

Reliability method is rarely used in the decision making of construction schemes, which is usually simply mentioned in three elements of the project in management references. In [13], reliability method is applied in the evaluation of the construction procedure [13].

In multiobjective optimization decision-making evaluation of projects, the relatively important degree of each evaluation index usually should be considered. The most direct and simple method expressing the important degree of each evaluation target is to give each target relevant weight. The entropy is a very ideal criterion to be applied for evaluating different decision-making processes. Applying the entropy principle to determine the weights of evaluation indexes has the scientific and the accuracy nature. In 1991, two Chinese scholars GU Changyao and QIU Wanhua firstly defined complex entropy and apply it in decision analysis. In 1994, QIU Wanhua also presents group decision-making complex entropy model [14].

In [15], the authors presented the evolution of concepts, an overview of research and applications pertaining to reliability in construction production, and the use of reserves, robust itineraries, and contingency of time and cost. It describes areas of management advisory systems in relation to the cycle of risk analysis [15].

In [16], a biobjective genetic algorithm was employed to solve the multiperiod network optimization problem, and a numerical example shows that the optimal coordination saves more than 50% of waste in system costs, compared to the worst-case scenario [16].

Making use of many existed studying results, this paper integrates engineering economics, risk and reliability theories, and information entropy theory to present a set of detailed engineering management decision methods of building construction projects combined with the concrete example. Presented detailed methods and steps can offer the reference for engineering management decision of building construction projects.

**Figure 1:**
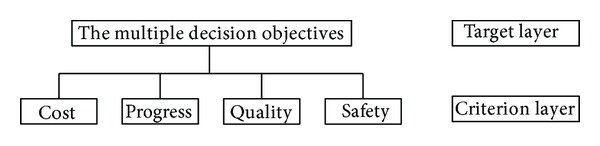
The multiobjective evaluation chart of building project construction schemes.

On the basis of summarizing and absorbing some existed references, this paper selected engineering cost, progress, quality, and safety as first-order criterion indexes, shown in Figure 1. For every first-order criterion index, further extended analysis calculation was done.

## 2. Calculating Cost of Building Construction Schemes

Combined with practical engineering experiences, the building construction cost of engineering projects includes direct cost and indirect cost. The direct cost also includes direct labor cost (*C*_1_), direct material cost (*C*_2_), direct mechanical cost (*C*_3_), and direct measure cost (*C*_4_). The indirect cost (*C*_5_) includes building construction stipulated expense and enterprise administration expense. The detail composition of building construction cost can be expressed by Table 1.

The total building construction cost can be calculated by

(1) $$\begin{array}{c} C=C_{1}+C_{2}+C_{3}+C_{4}+C_{5}. \end{array}$$

## 3. Calculating the Progress Score Value of Building Construction Schemes Combined with Reliability Theory

Combined with practical engineering experiences, this paper divided the whole building construction project engineering into 10 first-order progress segments; moreover, the first-order progress segment is divided into detailed second-order progress segments, as shown in Table 2. The progress score value of every second-order progress segment can be given out directly by domain experts. 

The total progress score value can be calculated by

(2) $$\begin{array}{c} R=\sum_{i=1}^{10}R_{i}, \end{array}$$

where *R*_*i*_ is the sum of progress score value of the *i* first-order progress segment.

For calculating *R*_*i*_, the authors make use of related knowledge in reliability theory. Considering that the progress relations of various second-order progress segments among goods transportation progress (*T*_8_) are parallel, so *R*_8_ can be calculated by

(3) $$\begin{array}{c} R_{8}=1-\left(1-R_{81}\right)\left(1-R_{82}\right)\left(1-R_{83}\right). \end{array}$$

For other *R*_*i*_ except for *R*_8_, the progress relation of various second-order progress segments are a series, so *R*_*i*_ except for *R*_8_ can be calculated by

(4) $$\begin{array}{c} R_{i}=\sum_{j=1}^{n_{i}}R_{ij}, \end{array}$$

where *n*_*i*_ is the number of second-order progress segments among the *i* first-order progress segment.

## 4. Calculating Quality Score Value of Building Construction Schemes Combined with Reliability Theory 

The authors divide the whole building construction project engineering into 7 first-order influence quality factor segments. Every first-order influence quality factor segment is divided into detailed second-order segments, as shown in Table 3.

For calculating the quality score value of every second-order influence quality factor segment, this paper divides them into two types; one type can be calculated by related reliability method, including collecting failure data, putting forward hypotheses by the frequency histogram, estimating parameters, and testing hypothesis. The other type can be calculated by the expert evaluation method.

Combined with practical engineering knowledge, the quality relations of various second-order quality segments included in every first-order quality segment are a series, so the quality score value of every first-order quality segment can be calculated by

(5) $$\begin{array}{c} A_{i}=\sum_{j=1}^{n_{i}}A_{ij}, \end{array}$$

where *n*_*i*_ is the number of second-order quality segments among the *i* first-order quality segment.

The total quality score value can be calculated by

(6) $$\begin{array}{c} A=\sum_{i=1}^{7}A_{i}. \end{array}$$

## 5. Calculating Safety Score Value of Building Construction Schemes

Factors affecting building construction safety mainly include direct factor and indirect factor. Direct factors include human factor, matter factor, and environment factor; indirect factors include management factor, and it is caused by three direct factors. This paper further analyzes three direct factors; the detailed composition of building construction safety influence factor is shown inTable 4.

In this paper, the safety score value can be calculated by

(7) $$\begin{array}{c} W_{i}=M_{i}\times P_{i}\times F_{i}, \end{array}$$

where *M* expresses the possibility risk degree index caused by unsafe factors in *i* safety factor, *P* expresses the probability risk degree index caused by *i* unsafe factor, and *F* expresses the produced result risk degree index after the accident in *i* safety factor. The value of three indexes can be obtained by experts according to Table 5.

The total quality score value can be calculated by

(8) $$\begin{array}{c} W=\sum_{i=1}^{3}W_{i}. \end{array}$$

## 6. Case Studies and Synthesis Computational Method Based on Integrated Information Entropy with Reliability Theory 

### 6.1. Case Analysis

Taking a building construction engineering project, for example, it is located in the third ring road east section of a city outskirt, has convenient traffic environment. There are residential buildings on the east, west, and north sides of this project; a Greenbelt Park is located in the south side of it. The total land area is 15 acres, the plot ratio is 2.1, and it is planned to be completed in one stage. The building engineering construction will begin on March 1, 2013; the planned construction period is 12 months.

The construction scheme 1 is described as follows. The expected period of engineering construction is 12 months. The month construction completed rate of this scheme, respectively, is 8%, 10%, 11%, 10 %, 8%, 8%, 10%, 11%, 8%, 6%, 6%, and 4%. The month construction progresses in winter and summer is slower than other months because of the effects of the natural environment.

In construction scheme 2, the expected period of engineering construction is 11 months. The month construction completed rate of this scheme, respectively, is 9%, 11%, 11%, 11%, 9%, 9%, 12%, 11%, 8%, 8%, and 7%.

In construction scheme 3, the expected period of engineering construction is 11 months. The month construction completed rate of this scheme, respectively, is 9%, 10%, 11%, 10%, 9%, 8%, 10%, 10%, 8%, 7%, 6%, and 2%.

In construction scheme  4, the expected period of engineering construction is 12 months. The month construction completed rate of this scheme, respectively, is 7%, 9%, 9%, 9%, 8%, 8%, 10%, 10%, 9%, 8%, 7%, and 6%.

According to Table 1 and formula (1) to calculate, respectively, the cost of 4 construction schemes, the result is shown in Table 6.

According to Table 2, formula (2), (3), and (4) to calculate, respectively, progress score value of 4 building construction schemes, the result is shown in Table 7.

According to Table 3, formula (5) and (6) to calculate, respectively, quality score value of 4 building construction schemes, the result is shown in Table 8.

According to Tables 4 and 5, and formula (7) and (8) to calculate, respectively, safety score value of 4 building construction schemes, the result is shown in Table 9.

According to above detailed calculating methods and steps, the result is shown as a calculated total value of 4 indexes including cost, progress, quality, and safety of 4 building construction schemes, as shown in Table 10.

### 6.2. Detailed Computing Steps of Entropy Weight

Regarding a multiobjective decision making problem that has *m* selected schemes and *n* evaluation indexes, detailed computing steps of entropy weight are as follows.

(1) Establishe evaluation index matrix including each evaluation index and corresponding evaluation value:

(9) $$\begin{array}{c} A={\left(a_{ij}\right)}_{m\times n}. \end{array}$$

(2) Standardize evaluation index matrix.

For the index that is “the bigger, the better,” the standardized value *r*_*ij*_ of the evaluation index can be calculated by

(10) $$\begin{array}{c} {r_{i}}_{j}=\frac{a_{ij}-\min a_{ij}}{\max a_{ij}-\min a_{ij}}. \end{array}$$

For the index that is “the smaller, the better,” the standardized value *r*_*ij*_ of the evaluation index can be calculated by

(11) $$\begin{array}{c} {r_{i}}_{j}=\frac{\max a_{ij}-a_{ij}}{\max a_{ij}-\min a_{ij}}. \end{array}$$

So the standardized decision-making matrix (*R* = (*r*_*ij*_)_*m*×*n*_, *r*_*ij*_ ∈ [0,1]) can be obtained.

(3) Calculate the entropy value of each evaluation index:

(12) Hj=−k∑i=1mfijln⁡fij (j=1,…,n)×(fij=rij(∑i=1mrij),k=1(ln⁡m)).

(4) Calculate the comprehensive feudatory degree *Z*_*i*_ of each evaluation object:

(13) $$\begin{array}{c} \omega_{j}=\frac{1-H_{j}}{n-\sum_{j=1}^{n}H_{j}}, \end{array}$$

(14) $$\begin{array}{c} Z_{i}={\left(r_{ij}\right)}_{m\times n}\omega^{T}. \end{array}$$

### 6.3. Calculations Combined with Case

Standardizes evaluation index matrix composed of value in Table 10. Cost and safety indexes are ones that is “the smaller, the better”, making use of a formula (11) to calculate. Progress and quality indexes are ones that is “the bigger, the better”, making use of a formula (10) to calculate. The standardization result is shown in Table 11”.

Making use of formula (12) and (13) to calculate the entropy value and entropy weight of 4 schemes, the result is shown in Table 12.

Calculates comprehensive feudatory degree *Z*_*i*_ of each evaluation scheme by formula (14) are shown as follows:

(15) Zi=(rij)m×mωT=[0.1360.6450.0010.550.0010.0320.4301.001.0001.0001.0000.670.2640.0010.2000.01][0.2190.2950.0870.400]=[0.440,0.447,0.869,0.080].

## 7. Conclusions

Based on the above calculation results, 4 building construction project schemes can be selected according to such sequence: Scheme 3 > Scheme 2 > Scheme 1 > Scheme  4. Generally, the optimization decision of building construction schemes is usually multiobjective optimization decision-making problem affected by many factors. This paper selects cost, progress, quality, safety as the four first-order evaluation indexes, and further deployment analyses of these indexes integrate engineering economics, risk and reliability theories, and information entropy theory to present a new evaluation optimization method for building construction projects based on integrated information entropy with the reliability theory combined with a case study. Presented detailed methods and steps can offer the reference for engineering management decision for the building construction projects.

## Figures and Tables

**Table 1:** The detailed composition of building construction cost.

The total building construction cost
Direct cost
Direct labor cost (*C*_1_)
Basic wage (*C*_11_)
Wage allowance (*C*_12_)
Auxiliary wage (*C*_13_)
Welfare expense (*C*_14_)
Labor protection expense (*C*_15_)
Direct material cost (*C*_2_)
Material initial cost (*C*_21_)
Material transportation miscellaneous cost (*C*_22_)
Transportation loss cost (*C*_23_)
Purchasing and storage cost (*C*_24_)
Material inspection and testing cost (*C*_25_)
Direct mechanical cost (*C*_3_)
Mechanical depreciation cost (*C*_31_)
Repair cost (*C*_32_)
Installing/removing and external transportation cost (*C*_33_)
Mechanical labor cost (*C*_34_)
Fuel power cost (*C*_35_)
Road toll and vehicle and vessel subsidy expenses (*C*_36_)
Direct measure cost (*C*_4_)
Environmental protection expense (*C*_41_)
Civilized construction expense (*C*_42_)
Safe construction expense (*C*_43_)
Temporary construction expense (*C*_44_)
Night construction expense (*C*_45_)
Large-scale mechanical equipment inside and out expense
(*C*_46_)
Concrete formwork expense (*C*_47_)
Scaffold expense (*C*_48_)
Equipment protection expense (*C*_49_)
Dewatering expense (*C*_410_)
Indirect cost (*C*_5_)
Construction stipulated expense (*C*_51_)
Enterprise administration expense (*C*_52_)

**Table 2:** The detailed segment composition of building construction progress.

Construction progress		
First-order progress influence factor segments	Second-order progress influence factor segments	Progress score value
Site leveling progress (*T*_1_)	Scene investigation progress (*T*_11_)	*R*_11_
	Removing obstacle progress (*T*_12_)	*R*_12_
	Calibrating range, setting benchmarks, and grid progress (*T*_13_)	*R*_13_
	Measuring elevation progress (*T*_14_)	*R*_14_
	Calculating earthwork cut and fill engineering quantities progress (*T*_15_)	*R*_15_
	Land leveling progress (*T*_16_)	*R*_16_
	Field compaction progress (*T*_17_)	*R*_17_

Ground foundation progress (*T*_2_)	Earth excavation progress (*T*_21_)	*R*_21_
	Cushion placement progress (*T*_22_)	*R*_22_
	Foundation placement progress (*T*_23_)	*R*_23_
	Foundation wall placement progress (*T*_24_)	*R*_24_
	Base pillar, ground ring beam progress (*T*_25_)	*R*_25_
	Backfilling earthwork progress (*T*_26_)	*R*_26_

Body engineering progress (*T*_3_)	Reinforcement engineering progress (*T*_31_)	*R*_31_
	Formwork engineering progress (*T*_32_)	*R*_32_
	Concrete engineering progress (*T*_33_)	*R*_33_
	Masonry engineering progress (*T*_34_)	*R*_34_

Roof engineering progress (*T*_4_)	Clean up, leveling progress (*T*_41_)	*R*_41_
	Processing insulation layer progress (*T*_42_)	*R*_42_
	Waterproofing and drainage progress (*T*_43_)	*R*_43_
	Setting qualified seaming progress (*T*_44_)	*R*_44_
	Ventilation, exhaust progress (*T*_45_)	*R*_45_

Decoration and fitment progress (*T*_5_)	Groundwater seepage test of bathroom and kitchen progress (*T*_51_)	*R*_51_
	Laying chisel trough and circuit pipeline progress (*T*_52_)	*R*_52_
	Sealing and burying trunk progress (*T*_53_)	*R*_53_
	Decoration and fitment progress of public wall (*T*_54_)	*R*_54_
	Installing doors and windows progress (*T*_55_)	*R*_55_

Water, electricity, and HVAC progress (*T*_6_)	Water supply and sewerage progress (*T*_61_)	*R*_61_
	Laying circuit progress (*T*_62_)	*R*_62_
	Installing heating progress (*T*_63_)	*R*_63_
	Water, heating, and electrical test progress (*T*_64_)	*R*_64_

Equipment installation progress (*T*_7_)	Installing elevator progress (*T*_71_)	*R*_71_
	Installing fire-fighting equipment progress (*T*_72_)	*R*_72_
	Installing emergency device progress (*T*_73_)	*R*_73_

Goods transportation progress (*T*_8_)	Goods transportation scheme 1 progress (*T*_81_)	*R*_81_
	Goods transportation scheme 2 progress (*T*_82_)	*R*_82_
	Goods transportation scheme 3 progress (*T*_83_)	*R*_83_

Environmental factor progress (*T*_9_)	Natural environment factor progress (*T*_91_)	*R*_91_
	Social environment factor progress (*T*_92_)	*R*_92_

Other factors progress (*T*_10_)	Construction design drawings change progress (*T*_101_)	*R*_101_
	Construction machinery, materials changes progress (*T*_102_)	*R*_102_
	Progress affected by weak supervision (*T*_103_)	*R*_103_
	Progress affected by supply failure of the municipal system (*T*_104_)	*R*_104_
	Progress affected by great political and social activities (*T*_105_)	*R*_105_

**Table 3:** The detailed quality influence factor segment composition of the building construction schemes.

Engineering quality		
First-order influence quality factor segments	Second-order influence quality factor segments	Quality score value
Site leveling quality segment (*Q*_1_)	Machinery performance influence quality segment (*Q*_11_)	*A*_11_
	Measuring tool influence quality segment (*Q*_12_)	*A*_12_
	Climate influence quality segment (*Q*_13_)	*A*_13_
	Worker influence quality segment (*Q*_14_)	*A*_14_

Ground and foundation quality segment (*Q*_2_)	Equipment performance influence quality segment (*Q*_21_)	*A*_21_
	Materials reliability influence quality segment (*Q*_22_)	*A*_22_
	Personnel quality influence quality segment (*Q*_23_)	*A*_23_
	Drawings reliability influence quality segment (*Q*_24_)	*A*_24_
	Construction plans influence quality segment (*Q*_25_)	*A*_25_
	Construction technology influence quality segment (*Q*_26_)	*A*_26_
	Construction environment influence quality segment (*Q*_27_)	*A*_27_

Main body engineering quality segment (*Q*_3_)	Construction machinery influence quality segment (*Q*_31_)	*A*_31_
	Construction technology influence quality segment (*Q*_32_)	*A*_32_
	Manager's quality influence quality segment (*Q*_33_)	*A*_33_
	Construction technique maturation influence quality segment (*Q*_34_)	*A*_34_
	Construction materials influence quality segment (*Q*_35_)	*A*_35_
	Construction worker technology influence quality segment (*Q*_36_)	*A*_36_
	Construction drawing reliability influence quality segment (*Q*_37_)	*A*_37_

Decoration and fitment engineering quality segment (*Q*_4_)	Decoration and fitment material influence quality segment (*Q*_41_)	*A*_41_
	Construction worker influence quality segment (*Q*_42_)	*A*_42_
	Constructor influence quality segment (*Q*_43_)	*A*_43_
	Construction equipment influence quality segment (*Q*_44_)	*A*_44_

Building roof quality segment (*Q*_5_)	Waterproof and thermal insulation material reliability influence quality segment (*Q*_51_)	*A*_51_
	Construction technology and procedure influence quality segment (*Q*_52_)	*A*_52_
	Working environment influence quality segment (*Q*_53_)	*A*_53_

Building water supply and drainage, heating quality segment (*Q*_6_)	Water supply and drainage, heating ventilating pipe influence quality segment (*Q*_61_)	*A*_61_
	Constructor technology level influence quality segment (*Q*_62_)	*A*_62_
	Construction technology influence quality segment (*Q*_63_)	*A*_63_

Building equipment and installation quality segment (*Q*_7_)	Equipment reliability influence quality segment (*Q*_71_)	*A*_71_
	Professional technology constructor influence quality segment (*Q*_71_)	*A*_72_

**Table 4:** The detailed composition of building construction safety influence factor.

Human safety factor (*S*_1_)	Matter safety factor (*S*_2_)	Environment safety factor (*S*_3_)
Not wearing security protection apparatus (*S*_11_)	Not setting safety protection (*S*_21_)	Construction natural conditions (*S*_31_)
Unsafe costume (*S*_12_)	Incorrect safety protection and dice marking (*S*_22_)	Narrow construction work surface (*S*_32_)
Wrong mechanical operation (*S*_13_)	Mechanical equipment being in the unsafe state (*S*_23_)	Disorderly construction yard (*S*_33_)
Ignoring safety warning (*S*_14_)	Mechanical equipment being in nonnormal state (*S*_24_)	Artificial lighting in the night is lacking (*S*_34_)
Using unsafe equipment (*S*_15_)	The material stacked in the unsafe state (*S*_25_)	Wrong operation process design (*S*_35_)
Body or spirit reason (*S*_16_)	Having harmful material (*S*_26_)	Bad ventilation (*S*_36_)
Rash advance operation (*S*_17_)		Improper protection for traffic line (*S*_37_)
Being in unsafe site (*S*_18_)		
Error handling of dangerous goods (*S*_19_)		

**Table 5:** Taking value of three risk degree indexes.

Possibility		Probability		Result	
Caused accident possibility	Taking the value of *M *	Caused accident probability	Taking the value of *P *	The result caused by accident	Taking the value of *F *
Sustainably occur	10	Very large possible occurrence	10	Extreme large accident, shutdown, and rectification	10
Often occur	8	Large possible occurrence	8	Very large accident, causing death	9
Occur for many times	6	Possible occurrence	6	Severe accident, having severe injury	7
Occur in a few times	5	Occur for once a while	5	Accident, having slight injury	5
Occur for very few times	3	Rare occurrence	3	Small accident, having minor injures	3
Basically not occur	1	Basically impossible	1	Very small accident, no injury	1

**Table 6:** The calculated cost value of 4 construction schemes (unit: ten thousand yuan).

	*C*_1_	*C*_2_	*C*_3_	*C*_4_	*C*_5_
Scheme 1	89.70	110.85	69.80	13.52	22.71
Scheme 2	76.80	121.90	73.75	13.62	22.89
Scheme 3	81.45	115.37	60.15	12.85	21.59
Scheme 4	79.45	118.69	70.23	13.42	22.54

**Table 7:** The calculated progress score value of 4 building construction schemes.

	Scheme 1	Scheme 2	Scheme 3	Scheme 4
*T*_11_	0.98	0.96	0.98	0.97
*T*_12_	0.92	0.93	0.94	0.95
*T*_13_	0.97	0.95	0.86	0.95
*T*_14_	0.87	0.86	0.92	0.98
*T*_15_	0.99	0.87	0.95	0.95
*T*_16_	0.96	0.91	0.89	0.88
*T*_17_	0.89	0.97	0.97	0.93
*T*_21_	0.95	0.94	0.97	0.94
*T*_22_	0.92	0.98	0.86	0.89
*T*_23_	0.93	0.91	0.94	0.93
*T*_24_	0.99	0.98	0.89	0.92
*T*_25_	0.98	0.92	0.99	0.89
*T*_26_	0.88	0.97	0.97	0.99
*T*_31_	0.98	0.87	0.95	0.91
*T*_32_	0.94	0.95	0.92	0.98
*T*_33_	0.97	0.88	0.92	0.87
*T*_34_	0.91	0.86	0.94	0.87
*T*_41_	0.89	0.91	0.91	0.92
*T*_42_	0.92	0.87	0.96	0.98
*T*_43_	0.89	0.89	0.88	0.96
*T*_44_	0.92	0.94	0.91	0.87
*T*_45_	0.97	0.85	0.90	0.96
*T*_51_	0.88	0.98	0.94	0.87
*T*_52_	0.85	0.91	0.87	0.91
*T*_53_	0.98	0.94	0.86	0.92
*T*_54_	0.89	0.95	0.86	0.86
*T*_55_	0.99	0.91	0.91	0.92
*T*_61_	0.86	0.88	0.87	0.96
*T*_62_	0.98	0.96	0.89	0.98
*T*_63_	0.92	0.88	0.90	0.92
*T*_64_	0.91	0.91	0.90	0.95
*T*_71_	0.90	0.93	0.88	0.95
*T*_72_	0.88	0.95	0.91	0.94
*T*_73_	0.92	0.86	0.90	0.85
*T*_81_	0.89	0.91	0.91	0.85
*T*_82_	0.92	0.88	0.94	0.88
*T*_83_	0.88	0.90	0.86	0.96
*T*_91_	0.86	0.93	0.96	0.99
*T*_92_	0.89	0.95	0.95	0.86
*T*_101_	0.89	0.87	0.96	0.88
*T*_102_	0.85	0.85	0.89	0.98
*T*_103_	0.93	0.94	0.98	0.91
*T*_104_	0.94	0.97	0.91	0.89
*T*_105_	0.98	0.88	0.93	0.86

**Table 8:** The calculated quality score value of 4 building construction schemes.

	Scheme 1	Scheme 2	Scheme 3	Scheme 4
*Q*_11_	0.97	0.92	0.96	0.90
*Q*_12_	0.93	0.91	0.98	0.93
*Q*_13_	0.99	0.91	0.97	0.96
*Q*_14_	0.92	0.94	0.98	0.96
*Q*_21_	0.93	0.97	0.97	0.98
*Q*_22_	0.95	0.93	0.94	0.99
*Q*_23_	0.94	0.99	0.91	0.93
*Q*_24_	0.91	0.97	0.96	0.92
*Q*_25_	0.91	0.94	0.96	0.96
*Q*_26_	0.94	0.96	0.96	0.98
*Q*_27_	0.92	0.97	0.93	0.90
*Q*_31_	0.98	0.97	0.97	0.91
*Q*_32_	0.91	0.98	0.92	0.92
*Q*_33_	0.95	0.90	0.97	0.93
*Q*_34_	0.92	0.92	0.91	0.97
*Q*_35_	0.95	0.91	0.91	0.97
*Q*_36_	0.91	0.96	0.96	0.90
*Q*_37_	0.95	0.93	0.98	0.95
*Q*_41_	0.94	0.98	0.98	0.92
*Q*_42_	0.92	0.97	0.97	0.93
*Q*_43_	0.92	0.94	0.98	0.95
*Q*_44_	0.93	0.94	0.93	0.96
*Q*_51_	0.90	0.92	0.91	0.94
*Q*_52_	0.96	0.96	0.95	0.91
*Q*_53_	0.94	0.98	0.96	0.96
*Q*_61_	0.97	0.94	0.91	0.93
*Q*_62_	0.90	0.94	0.92	0.97
*Q*_63_	0.96	0.98	0.96	0.94
*Q*_71_	0.92	0.92	0.93	0.98
*Q*_72_	0.90	0.93	0.92	0.94

**Table 9:** The calculated safety score value of 4 building construction schemes.

Schemes	Scheme 1			Scheme 2			Scheme 3			Scheme 4		
Risk degree indexes	*M*	*P*	*F*	*M*	*P*	*F*	*M*	*P*	*F*	*M*	*P*	*F*
*S*_11_	5	8	3	8	5	5	6	5	3	1	5	3
*S*_12_	8	3	2	4	1	7	5	8	2	5	7	2
*S*_13_	6	1	5	8	6	3	4	4	3	2	8	3
*S*_14_	8	1	1	7	6	4	8	4	2	9	2	3
*S*_15_	2	6	3	6	3	9	3	7	6	6	8	5
*S*_16_	5	4	5	4	3	3	1	5	7	7	3	1
*S*_17_	5	6	7	5	3	3	2	5	2	1	5	8
*S*_18_	5	5	9	7	6	4	4	7	7	5	5	5
*S*_19_	5	7	7	8	3	8	7	4	5	1	8	6
*S*_21_	6	5	4	5	2	3	5	6	5	3	7	9
*S*_22_	8	6	2	2	9	1	1	3	1	5	3	8
*S*_23_	8	9	4	9	4	7	4	1	9	5	8	4
*S*_24_	8	6	5	2	1	5	4	9	4	9	1	9
*S*_25_	2	8	4	8	7	2	8	8	4	8	1	3
*S*_26_	6	6	1	9	7	2	3	7	6	8	6	3
*S*_31_	5	8	3	8	5	4	2	6	8	3	4	9
*S*_32_	5	6	3	8	3	7	6	7	2	9	7	7
*S*_33_	5	8	1	6	2	1	5	4	9	2	8	9
*S*_34_	1	3	8	7	2	8	6	8	2	2	1	5
*S*_35_	8	3	2	2	3	6	5	6	9	7	4	9
*S*_36_	1	5	9	8	3	2	1	2	5	7	7	8
*S*_37_	6	7	7	3	1	2	5	5	8	8	5	4

**Table 10:** The calculated total value of 4 indexes including cost, progress, quality, and safety of 4 building construction schemes.

	Cost	Progress	Quality	Safety
Scheme 1	306.58	0.192	0.233	2527.00
Scheme 2	308.96	0.173	0.291	2233.00
Scheme 3	291.40	0.203	0.368	2450.00
Scheme 4	304.33	0.172	0.260	2886.00

**Table 11:** The standardization result of 4 building construction schemes based on 4 decision indexes.

	Cost	Progress	Quality	Safety
Scheme 1	0.136	0.645	0.001	0.55
Scheme 2	0.001	0.032	0.430	1.00
Scheme 3	1.000	1.000	1.000	0.67
Scheme 4	0.264	0.001	0.200	0.01

**Table 12:** The calculated entropy value and entropy weight of 4 schemes.

Indexes	Cost	Progress	Quality	Safety
Entropy value (*H*_*i*_)	0.678	0.567	0.873	0.413
Entropy weighs (*ω*_*i*_)	0.219	0.295	0.087	0.400

## References

[1] Xu Y, Wang Y, Yao B (2008). Construction project stakeholder collaboration group decision making based on entropy theory. *Chinese Journal of Management Science*.

[2] Feng Y, Shi K (2009). Optimum decision-making of deep foundation pit construction project based on the least variance priority method. *Building Science*.

[3] Tian B (2009). *Management Science in Engineering Project*.

[4] Chen Y, Peng X (2007). Method of analytical hierarchy process making for decision on construction scheme. *Journal of Zhengzhou University of Light Industry (Natural Science)*.

[5] Chen J (2010). On construction scheme selected based on value engineering. *Shanxi Architecture*.

[6] Gao S, Du H (2003). Study on comprehensive evaluation method about engineering construction scheme based on Grey Correlation Degree. *Coal Mine Engineering*.

[7] Feng Y (2006). Optimum decision-making of construction project based on the least variance priority method. *Mathematics in Practice and Theory*.

[8] Oiu W (2001). *Management Decision and Application Entropy*.

[9] Wang J, Liu E (2004). Analysis of time-cost-quality tradeoff optimization in construction project management. *Journal of Systems Engineering*.

[10] Touboul J (2011). Projection pursuit through relative entropy minimization. *Communications in Statistics*.

[11] Liu Q, Yang Q (1997). The control of cost, duration, ‘quality and safety in project management of construction’. *Journal of Ningxia Institute of Technology (Natural Science)*.

[12] Lu N, Shi Y, Gao X, Li W, Liao X (2006). Calculation method of construction working procedure. *Journal of Xi’an University of Architecture & Technology (Natural Science Edition)*.

[13] Qiu W (1995). An entropy model on group decision system. *Control and Decision*.

[14] Ma L, Gao Q (2010). Analysis of organizational structure for human resource management department based on structure-entropy model. *Industrial Engineering Journal*.

[15] Turskis Z, Gajzler M, Dziadosz A (2012). Reliability, risk management, and contingency of construction processes and projects. *Journal of Civil Engineering and Management*.

[16] Oh J, Kim H, Park D (2011). Bi-objective network optimization for spatial and temporal coordination of multiple highway construction projects. *KSCE Journal of Civil Engineering*.

